# Plant-Derived Exosomes Deliver Theranostic Molecules to Target Cells

**DOI:** 10.3390/nano16120744

**Published:** 2026-06-14

**Authors:** Davide Mizzoni, Rossella Di Raimo, Antonella Aloi, Giuseppina Poppa, Vincenza Dolo, Mariantonia Logozzi, Stefano Fais

**Affiliations:** 1 Department of Oncology and Molecular Medicine, Istituto Superiore di Sanità, Viale Regina Elena 299, 00161 Rome, Italy; davide@exolabitalia.com (D.M.); rossella@exolabitalia.com (R.D.R.); 2 ExoLab Italia, Tecnopolo D’Abruzzo, 67100 L’Aquila, Italy; antonella.aloi@exolabitalia.com; 3 Department of Clinical Medicine, Public Health, Life and Environmental Sciences, University of L’Aquila, 67100 L’Aquila, Italy; giuseppina.poppa@univaq.it (G.P.); vincenza.dolo@univaq.it (V.D.)

**Keywords:** plant, exosomes, drug delivery, acridine orange, anti-tumor effect

## Abstract

More specific targeted drug delivery systems with low immunogenicity and toxicity are expected to increase the efficacy of therapeutic molecules. The extracellular nanovesicles have been introduced as natural delivery systems for therapeutic molecules. More recently, considerable interest has emerged in plant-derived exosomes and their natural commitment to deliver molecules of various origins. Acridine Orange (AO) is an acidophilic dye with a strong tumoricidal action following excitation with a light source at 466 nm, but its clinical use is limited by the potential systemic toxicity. In this study we investigated the ability of exosomes from *Citrus Sinensis* to be successfully uploaded with AO (Exo-AO). We also studied the ability of the exosomes to be uploaded into target cells, as compared to the free molecules. We found that AO was efficiently uploaded into exosomes through electroporation. In fact, Exo-AO entered into the target cells significantly better than the free molecules, leading to both a marked intracellular AO delivery into target cells and an increase in the cytotoxic effect after excitation under a fluorescence microscope. This study shows a promising new approach for a more effective and less toxic drug delivery through the use of plant-derived extracellular nanovesicles.

## 1. Introduction

In recent decades many attempts have been made in order to improve the delivery of therapeutic molecules to the sites where a disease is developing. This was because therapeutic molecules have a very narrow medical indication with overly often significant systemic side effects. There are many factors that may hamper the efficacy of drugs that have shown a significant effect in in vitro testing. These factors include all the mechanisms that define the “bioavailability”, such as drug transport through the blood stream, the cellular mechanism belonging to the scavenging framework, and the specific factors that define the tissue that should be the major target of the drug. Acridine Orange (AO) may represent a prototype of molecules that behave both as a tracer and a therapeutic molecule. In fact, AO has different fluorescence; when it is illuminated with blue light (450–490 nm), it is visualized as green fluorescence (emission: 515–565 nm), while if the illumination is switched to the green light (546 nm) mode, AO is detected as red fluorescence (emission: 590 nm) [1,2,3,4]. AO has the property of being activated by radiation and by a specific wavelength (466 nm) (blue light) that results in a tumoricidal effect. These properties have been investigated both in vitro and in vivo, showing that AO could be used to detect (upon excitation from a proper light source) tumors, metastases and residual disease after surgical excision [5,6]. The first preclinical investigations prompted clinicians to take advantage of the preferential accumulation of AO in the acidic microenvironment of musculoskeletal tumors to detect minimal residual disease, thus allowing limb-sparing procedures. The same group has more recently described the successful outcome of patients with advanced high-grade sarcoma, rhabdomyosarcoma and musculoskeletal sarcoma, treated with surgery and adjuvant therapy with AO excited by radiation and photodynamic therapy. The limiting factor of AO is its potential systemic toxicity, which has so far confined its application to local therapy, usually by topic administration. There is therefore the need for novel strategies to implement the use of AO in clinical practice and to allow the systemic administration, thus allowing the systemic treatment [6].

The use of nanoparticles to deliver molecules of various origins to target tissues has been a central issue in medical research. The aim was of course to increase the efficacy and reduce the unwanted side effects of each therapeutic molecule. The early idea was to build synthetic nanoparticles that enveloped the therapeutic molecules in order to avoid their degradation before reaching the target site. The artificial nanoparticles included polymer nanoparticles, solid lipid nanoparticles (SLNs), crystal nanoparticles, and liposomes. These artificial nanoparticles are used to encapsulate a series of molecules including proteins, phenolic peptides, and antibodies, but also siRNA, RNA, and DNA. However, a preliminary problem was always that the artificial nanoparticles are obtained by chemical synthesis that limits their clinical application due to the potential in vivo toxicity. Moreover, the industrial scalability is limited and the production costs too often exceed the forecast.

In recent decades scientific research is proposing the use of a family of extracellular vesicles (EVs), that are naturally released from cells of all living beings, as the most promising delivery system for a wide spectrum of molecules [7,8,9,10,11,12,13,14].

We have shown that extracellular vesicles (EVs) released by human normal peripheral blood mononuclear cells were able to deliver both chemical molecules and gold nanoparticles, and to transfer them to target cells where they, within EVs, are retained longer than the free molecules. However, very recently, the FDA has limited the use of human and animal derived EVs due to the potential danger for their role in scavenging toxic molecules. Moreover, it is hard to think about the industrial use of both human and animal EVs.

For this reason, in the last five years, research on plant-derived extracellular vesicles (PDEVs) has been raising great interest for their use as the ideal drug delivery system for therapeutic molecules. Previous investigation has compared PDEVs to synthetic nanoparticles (e.g., liposomes) and mammalian/human cell-derived extracellular vesicles (EVs) in also delivering anticancer drugs [15]. PDEVs are isolated from different edible sources, such as fruits and vegetables, starchy roots and tubers, nuts and seeds, and fresh and dried plants. PDEVs include nanovesicles, also called exosomes, with sizes ranging between 30 and 150 nm, that look ideal for drug delivery. In fact, previous studies have shown that PDEVs can also deliver a series of molecules, including chemotherapeutics, theranostic molecules, antibodies and nanoparticles, due to their scavenging activity in our organism [7,8,9,10,11,12,13,14,16,17]. PDEVs have shown a series of relevant advantages, such as high level of biocompatibility and low immunogenicity (being generally taken as daily foods). However, probably the most intriguing and promising advantage is that PDEVs may act as both potential therapeutic agents, depending on their natural content, and a natural delivery system for external molecules [18]. PDEVs may be uploaded with a broad range of molecules including drugs, nucleic acids, bioactives and proteins. All the alternative approaches, including human EVs and artificial nanoparticles, did not show comparable properties [15,16,17,18].

PDEVs from organic farming seem to represent one of the most promising tools for drug delivery because they do not contain the pesticides and microbicides commonly used in intensive agriculture, but also because of their more active content [19].

In this study we investigated the ability of exosomes from *Citrus Sinensis* to be successfully uploaded with the theranostic molecule Acridine Orange (AO) (Exo-AO). We also studied the uptake of Exo-AO by target cells, as compared to the free molecules. We found that the AO was efficiently uploaded into exosomes and that the Exo-AO made AO enter within the target cells significantly better than the free molecule. Moreover, the Exo-AO, following light excitation, was more cytotoxic against the target cells as compared to the free molecule.

## 2. Materials and Methods

### 2.1. Isolation of Plant-Derived Exosomes

Fruits were sourced from Italian farms with organic certification. After collection, all the samples were processed individually. They were washed with water and sodium bicarbonate sequentially and peeled for extraction through a juice extractor. Juices were stored at −80 °C for subsequent analysis. The procedure was followed according to a previously reported method [19].

Briefly, the extract was filtered through 1 mm filters, followed by differential centrifugation at 500× *g* for 10 min; the supernatants were filtered with 100 µm filters and serially centrifugated at 2000× *g* for 20 min to eliminate cell debris and then at 15,000× *g* for 30 min. The supernatants were filtered through 100 µm filters and subsequently ultracentrifuged in a Sorvall WX Ultracentrifuge Series (Thermo Fisher Scientific, Waltham, MA, USA) at 110,000× *g* for 1 h 30 min to collect the nanovesicles. The obtained exosome pellet was resuspended in PBS 1× and stored at +4 °C until further use. An aliquot of the exosomes was used for the analysis of concentration and dimensions.

### 2.2. Nanoparticle Tracking Analysis

Size distribution and concentration of extracellular vesicles in liquid suspension were analyzed through Nanoparticle Tracking Analysis (NTA) from Malvern (NanoSight NS300, Salisbury, UK). For each measurement, five videos of typically 60 s duration were taken, and data were analyzed using the NTA 3.4 software (Malvern Instruments, Malvern, UK). The Brownian motion of each particle was tracked using the Stokes–Einstein equation: D° = kT/6πηr, where D° is the diffusion coefficient, kT/6πηr = f_0_ is the frictional coefficient of the particle, and for the special case of a spherical particle of radius r moving at a uniform velocity in a continuous fluid of viscosity η, k is Boltzmann’s constant, and T is the absolute temperature.

### 2.3. Zeta Potential Measurements

The zeta potential of plant-derived exosomes was determined by Zetasizer Lab (Alfa test, Malvern Instruments, UK). The measurement of this parameter provides useful information on colloidal stability and electrostatic interactions of vesicles in suspension. Data processing was performed using the ZS XPLORER software (version 1.3 14.7, Malvern Instruments, UK). The stability of the dispersion is correlated with the absolute value of the zeta potential: values in the range of ±20–30 mV are generally considered indicative of high dispersion stability.

### 2.4. Phospholipid Assay Kit

The Phospholipid Assay Kit was selected for this study because it enables a simple, direct, and high-throughput quantification of choline-containing phospholipids in biological samples. The assay is based on the enzymatic hydrolysis of phospholipids, including lecithin, lysolecithin, and sphingomyelin, leading to the release of choline. The released choline is subsequently oxidized by choline oxidase, generating hydrogen peroxide, which reacts with a specific probe to produce a colorimetric signal (570 nm). In order to evaluate the phospholipid content in exosomes, a commercially available Phospholipid Assay Kit (MAK122, Sigma-Aldrich, USA) was used. Briefly, samples were plated in 96-well plate, a reaction mix was added to each well and the plate was incubated for 30 min at room temperature, protected from light. After the incubation, the absorbance was measured at 570 nm using a Microplate Spectrophotometer (Agilent BioTek Epoch Microplate Spectrophotometer, BioTek, St. Louis, MO, USA) and the results were interpolated in the calibration curve, following manufacturer’s instructions.

### 2.5. Enzyme-Linked ImmunoSorbent Assay (ELISA)

In order to evaluate the expression of the typical plant exosome markers, exosomes were analyzed through an ELISA. Briefly, 10 µg/well of exosomes were resuspended in coating buffer (15 mM Na2CO3, 35 mM NaHCO3, pH 9.6) and coated on MaxiSorp High Protein-Binding plates at 4 °C overnight. Coated wells were washed three times with PBS +0.5% Tween 20 (PBST) and blocked in a solution composed by PBS and Bovine Serum Albumin Fraction V (10735078001, Sigma-Aldrich, USA) for one hour at room temperature. Then, primary antibody TET8 1:1000 (PHY1491S, PHYTOAB, USA) was added in each well and incubated for one hour at room temperature under agitation. After three washes with PBST, the secondary antibody Goat Anti-Rabbit IgGH&L (HRP) 1:2500 (PHY6000, PHYTOAB, USA) was added to the well for one hour under agitation. The plate was then incubated with TMB substrate (T0440, Sigma-Aldrich, USA) for 15 min and after the addition of the stop solution (S5814 Sigma-Aldrich, USA), the absorbances were read using a Microplate Spectrophotometer (Agilent BioTek Epoch Microplate Spectrophotometer, BioTek, CA, USA) [20,21].

### 2.6. Transmission Electron Microscopy (TEM)

The plant-derived extracellular vesicles (PDEVs) were resuspended in phosphate-buffered saline (PBS), and a small volume of the sample was deposited on 200-mesh carbon-coated copper grids and allowed to adhere. The grids were then fixed with 2% glutaraldehyde (Electron Microscopy Sciences, Hatfield, PA, USA) in PBS. After fixation, the samples underwent three washing steps with milliQ water to remove excess fixative and unwanted residues. To enhance contrast, a negative staining procedure was performed using a 2% phosphotungstic acid solution. TEM images were acquired using a Philips CM 100 Electron Microscope at an accelerating voltage suitable for optimal resolution.

### 2.7. Electroporation Procedure

Plant-derived exosomes were isolated from organic fruits (*Citrus paradisi*, *Citrus limon* (L.), *Citrus sinensis*) obtained from certified Italian farms. Isolation was performed following protocol, based on serial centrifugation and ultracentrifugation, in accordance with internationally accepted EV purification guidelines [22]. The isolated exosomes were resuspended in sterile 1× phosphate-buffered saline (PBS, pH 7.4, without Ca^2+^ and Mg^2+^). Exosomes (10^10^, 10^11^, 2 × 10^11^ vesicles), resuspended in 1000 µL of PBS, were transferred into sterile 0.4 cm electroporation cuvettes. Acridine Orange (AO) was added at concentrations ranging from 0.1 to 100 µg/mL. Electroporation was performed using the following pulse conditions: with a first pulse of 300 V with a duration of 100 µs, a pause for 1000 ms and then a series of eight pulses of 80 V with a duration of 30 ms each. According to the patented loading strategy adopted in this study, the initial high-voltage pulse was intended to induce transient membrane destabilization, whereas the subsequent lower-voltage pulse train was used to facilitate transmembrane molecular transfer under controlled permeabilization conditions. Immediately after pulse delivery, electrical impedance (Ω) was recorded as a dynamic physicochemical parameter associated with the electrical behavior of the vesicular system during electroporation. Impedance measurements were used to monitor electroporation-associated membrane perturbation and loading conditions and were not intended to represent direct evidence of specific dielectric phase transitions. Following electroporation, samples were ultracentrifuged at 110,000× *g* for 90 min to remove non-associated AO. The resulting pellet containing AO-loaded exosomes was resuspended in PBS, whereas the supernatant was retained for control analyses. Samples were allowed to stabilize prior to ultracentrifugation in order to minimize transient electroporation-associated membrane perturbations. Loaded exosomes were characterized using a Perkin-Elmer LS-50B microplate spectrofluorimeter (Waltham, MA, USA). AO loading was quantified by fluorescence analysis using excitation at 466 ± 5 nm and emission at 525 ± 5 nm. Final AO concentrations were calculated using calibration curves generated in parallel under identical experimental conditions. As the control sample, we measured the signal of unloaded exosomes, which was subtracted from the loaded ones in order to eliminate the background signal. All experiments were run in triplicate wells and repeated at least twice.

### 2.8. Impedance Measurements

The complex impedance (Ohm, Ω) of the system was measured immediately following pulse application. Samples were dispersed in a sterile 0.4 cm-gap electroporation cuvette. All measurements were performed at a controlled temperature of 25 °C to mitigate potential artifacts arising from Joule thermal dissipation. All experiments were run in triplicate wells and repeated at least twice. Actually, impedance was used exclusively as an indirect, label-free physical readout of the electroporation process.

### 2.9. Quantification of Loading Efficiency

Loading Efficiency (LE%) of Acridine Orange (AO) within plant-derived exosomes was evaluated through fluorometric analysis. Following electroporation, samples were ultracentrifuged at 110,000× *g* for 90 min to separate AO-loaded exosomes from the non-encapsulated dye. The pellet containing the loaded exosomes was resuspended in PBS, while the supernatant was collected to quantify the amount of free AO. As a preliminary step, a calibration curve obtained through the use of increasing concentrations of AO (going from 0.1 to 100 µg/mL) was performed. Fluorescence measurements were performed using a microplate spectrofluorimeter Perkin-Elmer LS-50B (Waltham, MA, USA). The auto-fluorescence background generated by uncharged exosomes was deducted from the overall fluorescence. All the experiments were carried on using plates for fluorescence using light at the wavelength of 466  ±  5 nm in excitation and 525  ±  5 nm filter in emission. The experiment was repeated for six times in duplicate. A calibration curve generated with known AO concentrations was used to interpolate the amount of encapsulated dye. All experiments were run in triplicate wells and repeated at least twice. Loading Efficiency (LE) was calculated according to the formula:

LE% = (amount loaded/total initial amount) × 100

All experiments were performed with 6 biological replicates, each measured in triplicate technical wells.

### 2.10. Cell Culture and Treatments

Human melanoma cells (Mel501 line) were cultured in RPMI 1640 medium supplemented with 10% Fetal Bovine Serum (FBS), 2 mM L-glutamine, and 1% penicillin/streptomycin, at 37 °C in a 5% CO_2_ humidified atmosphere. For uptake experiments, cells were seeded in 24-well plates at a density of 2 × 10^4^ cells/well and allowed to adhere for 24 h. Treatments were performed as follows: (i) unloaded *Citrus sinensis*-derived exosomes (Exo CTR); (ii) free Acridine Orange (AO) at a concentration of 200 ng/mL; and (iii) *Citrus sinensis*-derived exosomes loaded with AO (Exo-AO) at an equivalent dose of 200 ng/mL. Cells were incubated with the treatments for 24 h in a serum-free medium to eliminate interference from exogenous bovine-derived vesicles. To induce cytotoxicity, cells were exposed to blue light irradiation (466  ±  5 nm in excitation and 525  ±  5 nm filter in emission) for 10 s using the Optika microscope (IM-5FLD, Optika Microscopes, Bergamo, Italy). As the control sample we used the unloaded exosomes to eliminate the background signal. All experiments were run in triplicate wells and repeated at least twice. Cell Death was assessed using the Trypan Blue exclusion assay. Briefly, the culture medium was collected and combined with the trypsinized cells, then centrifuged to obtain a concentrated cell suspension containing both viable and dead cells. Cell counting was subsequently performed using a Neubauer chamber according to the standard procedure. Dead cells were expressed as a percentage of the total cell number.

### 2.11. Confocal Laser Scanning Microscopy (CLSM)

After the treatment, images were acquired using an inverted microscope (Nikon Ti-E) equipped with a confocal spectral imaging system (Nikon D Eclipse C1si) using a (Nikon) PlanApo objective 60× oil (numerical aperture 1.4). Acridine Orange fluorescence was excited using a 488 nm Argon laser, with emissions collected in the 495–550 nm range (green) to visualize the monomeric form of the dye. Phase-contrast images were also acquired to visualize the cell morphology. Differential Interference Contrast (DIC) was utilized to provide structural context and assess cell morphology. All acquisition parameters (laser power, gain, and offset) were kept constant across all experimental groups to ensure rigorous comparative quantification. Images recorded have an optical thickness of 0.20 µm and were analyzed by the 2023 version of C1-LCSI EZ-C1 software for spectral analysis. The signal from the fluorescent probe was taken in sequential scanning mode; several fields were analyzed for each condition, and representative results are shown.

### 2.12. Statistical Analysis

Results are reported as the means ± standard deviation (SD), and calculations were performed using GraphPad Prism software (Software version 8.0.2, USA). An unpaired *t*-test (Student’s *t*-test), one-way ANOVA Bonferroni and two-way analysis (Two-way ANOVA) were applied to analyze the results. Statistical significance was set at *p* < 0.05.

## 3. Results

### 3.1. Characterization of Plant-Derived Exosomes

In order to study the most suitable exosomes for the delivery of Acridine Orange we choose three different *Citrus* sources: grapefruit (*Citrus paradisi*), lemon (*Citrus limon*) and orange (*Citrus sinensis*). After the fruit extraction, the juices underwent differential centrifugation and ultracentrifugation, and the obtained exosomes were characterized through NTA, Zeta potential and phospholipid quantification. As summarized in Table 1, all three samples showed sizes included in the typical size of extracellular vesicles, with peaks at 164.0 ± 15.4, 169.9 ± 9.02 and 158.8 ± 18.7 respectively for grapefruit, lemon and orange.

**Figure 1:**
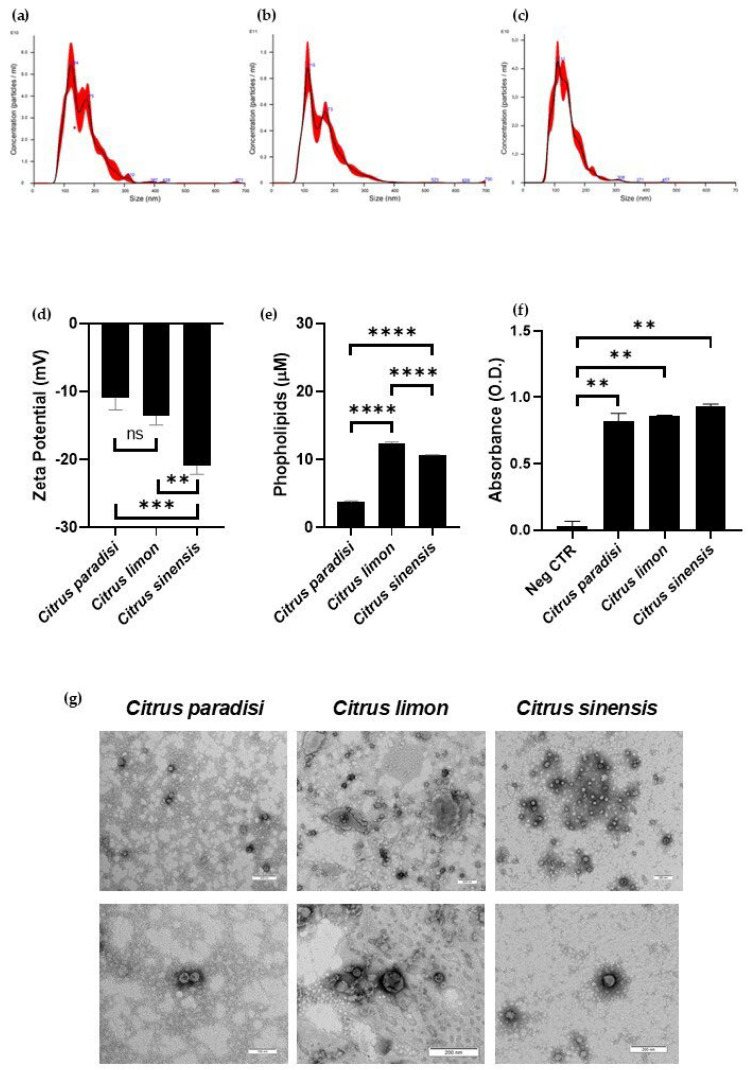
**Characterization of Plant-derived Exosomes.** Dimensional distribution of exosomes isolated from (**a**) grapefruit (*Citrus paradisi*), (**b**) lemon (*Citrus limon*) and (**c**) orange (*Citrus Sinensis*); (**d**) zeta potential quantification, (**e**) quantification of phospholipids, (**f**) ELISA, (**g**) morphological characterization through TEM. Upper panel of TEM analysis shows images acquired at lower magnification, lower panel at higher magnification. Scale bars: 200 nm for all images, except for the right grapefruit image, where the scale bar is 100 nm. Data are expressed as mean ± SD. * *p* < 0.05, ** *p* < 0.01, *** *p* < 0.001, **** *p* < 0.0001.

Figure 1a–c show the size distribution of exosomes from grapefruit (*Citrus paradisi)*, lemon (*Citrus limon)* and orange (*Citrus sinensis*) respectively, analyzed through NTA. Data are consistent with the typical distribution of plant-derived exosomes. In order to have a more complete characterization and a measure of their stability, zeta potential was analyzed and data are summarized in Figure 1d. Specifically *Citrus sinensis*-derived exosomes showed a more stable zeta potential, with values equal to −20.9 ± 1.9 mV compared to *Citrus paradisi*-derived exosomes (−10.9 ± 1.7 mV, *p* < 0.001), and *Citrus limon*-derived exosomes (−13.58 ± 1.4 mV, *p* < 0.01). As the membrane phospholipidic asset plays a crucial role in vesicle stability as their uptake in recipient cells, we performed a colorimetric assay in order to measure their phospholipid content. Starting from the same amount of exosomes (10^10^ vesicles), we found a detectable and measurable phospholipidic content, with a concentration of 3,76 ± 0,14 µM in grapefruit, 12.4 ± 0.2 in lemon and 10.7 ± 0.1 µM in *Citrus sinensis*-derived exosomes.

To further characterize the extracellular vesicles, samples derived from *C. paradisi*, *C. limon*, and *C. sinensis* were analyzed by ELISA to detect the presence of typical plant exosome markers. As shown in Figure 1f, all samples exhibited a significantly higher signal compared with the negative control, confirming the presence of TET8 in the analyzed plant-derived exosomes.

Furthermore, the typical exosomal morphology was investigated by TEM analysis (Figure 1g). The upper panels show images at lower magnification, whereas the lower panels display higher-magnification images. The images demonstrated that the isolated exosomes exhibited the characteristic morphology of extracellular vesicles, including a round cup-shaped structure surrounded by a membrane.

Taken together, these findings demonstrate that our extracellular vesicle preparations exhibit characteristics fully consistent with typical exosome populations, along with strong stability, as confirmed by both zeta potential measurements and phospholipid content analysis.

### 3.2. Dielectric Response and Electroporation-Induced Phase Transitions in Citrus Sinensis-Derived Exosomes

In this set of experiments, we used *Citrus Sinensis*-derived exosomes because, compared to the exosomes derived from lemon and grapefruit, they exhibited greater colloidal stability (a higher absolute zeta potential value), ideal for subsequent loading analyses. To optimize electroporation, we monitored the physical state of the vesicular membrane. The efficiency of electro-mediated loading was investigated by analyzing the dielectric response of *Citrus Sinensis*-derived exosomes (Exo) as a function of Acridine Orange (AO) concentration (0–100 µg/mL) and vesicle concentration (10^10^, 10^11^, 2 × 10^11^ exosomes/mL).

Vesicle-free controls were not included in this set of experiments inasmuch as they focused on loading efficiency and to provide proof of concept, rather than a biophysical mechanism.

**Figure 2:**
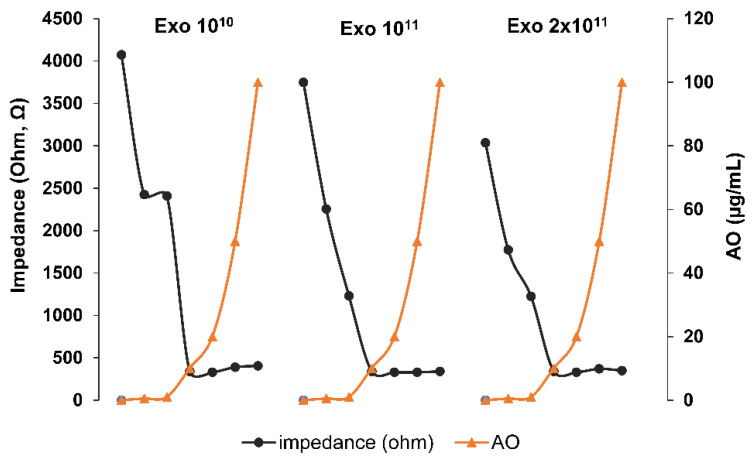
**Impedance dynamics and stability of electro-mediated loading.** Multi-panel dual Y-axis plots showing the biophysical and statistical analysis of *Citrus Sinensis*-derived exosomes (Exo) (n = 3 independent biological replicates). The three panels correspond to different vesicular numerical densities: 10^10^, 10^11^ and 2 × 10^11^ Exo/mL. The primary Y-axis (left, black line) reports the system impedance (Ohm, Ω); the secondary Y-axis (right, orange line) reports the loaded concentration of Acridine Orange (AO, µg/mL). A non-linear asymptotic dielectric phase transition correlated with cargo concentration is observed. Error bars are omitted for clarity, given the drastic reduction in variance observed at saturation regimes exceeding 10 µg/mL of AO, as confirmed by Two-way ANOVA analysis.

Complex impedance data (Ohm, Ω) revealed a distinct electrical phase transition correlated with solute (AO) concentration (Figure 2). In control samples (0 µg/mL), the system exhibited high basal impedance values (3.035–4.075 Ω), characterized by significant intrinsic variance [19,20]. The introduction of AO at sub-micromolar concentrations (0.5–1 µg/mL) triggered a gradual reduction in system resistance. However, a sharp collapse in impedance was observed within the 1 e 10 µg/mL range, subsequently stabilizing into an asymptotic plateau (330–400 Ω) across all tested vesicular densities. Two-way ANOVA confirmed that AO concentration exerted a dominant effect on system conductance (*p* < 0.0001), while exosome density modulated the stability of the response (*p* < 0.01). Notably, the standard deviation underwent a drastic contraction as AO concentration increased (from 143 to 13 Ω in the 10^10^ Exo/mL group). This indicates that the electroporation process and subsequent drug loading reduced the dielectric heterogeneity of the sample, driving the system toward a reproducible physical state. The change in impedance is the label-free diagnostic parameter that confirms successful permeabilization. We would like to emphasize that impedance was used with the only aim being to provide an indirect, label-free physical readout of the electroporation process.

### 3.3. Electroporation of Acridine Orange in Citrus Sinensis-Derived Exosomes

**Figure 3:**
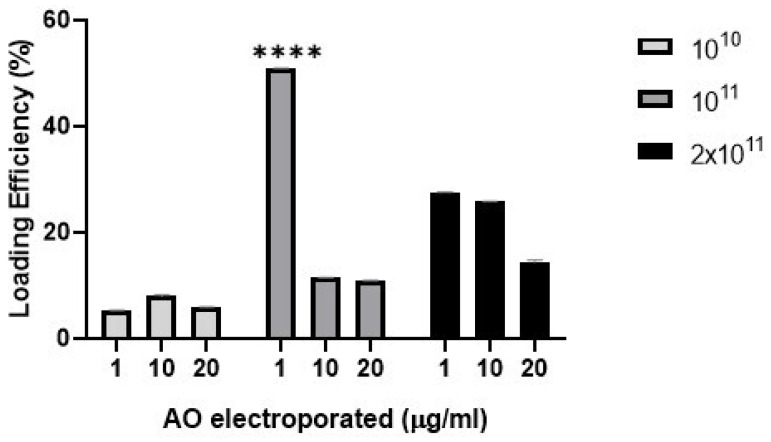
**Quantification of Acridine Orange (AO) encapsulated in *Citrus Sinensis*-derived exosomes**. 10^10^, 10^11^ and 2 × 10^11^ *Citrus Sinensis*-derived exosomes were electroporated with 1, 10 and 20 µg/mL of AO and the fluorescent signals were analyzed through a microplate fluorometer. Data are expressed as mean ± SD. * *p* < 0.05, ** *p* < 0.01, *** *p* < 0.001, **** *p* < 0.0001.

Based on the dielectric phase transition observed via impedance measurements, which identified the 1–20 µg/mL range as the most dynamic for membrane permeabilization, subsequent quantitative fluorometric analyses were focused on these concentrations to determine the optimal Loading Efficiency (LE%). Concentrations exceeding 20 µg/mL were excluded from further loading quantification as they yielded no significant further reduction in system impedance, suggesting a physical saturation of the exosomal loading capacity. Therefore, several concentrations of *Citrus Sinensis*-derived exosomes (10^10^,10^11^ and 2 × 10^11^ vesicles) were electroporated with 1, 10 and 20 µg/mL of Acridine Orange, and the fluorescence intensity was detected using a microplate fluorometer. A calibration curve with a known concentration of AO was used to interpolate and define the AO encapsulated in the vesicles. As reported in Figure 3, there were significant differences in encapsulation efficiency, with the highest encapsulation efficiency electroporating 1 µg/mL of Acridine Orange in 10^11^/mL exosomes (about 51% of encapsulation efficiency). Under the optimal loading condition (1 µg/mL AO and 10^11^ Exo/mL), the encapsulation efficiency reached approximately 51%, corresponding to about 0.51 µg of AO. For that reason, these conditions were used for subsequent experiments.

### 3.4. Evaluation of Acridine Orange Uptake

**Figure 4:**
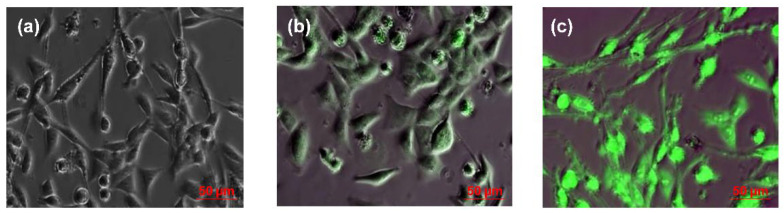
**Confocal laser scanning microscopy analysis of Acridine Orange (AO) uptake in Mel501 human melanoma cells.** (**a**) Mel501 cells treated with unloaded *Citrus Sinensis*-derived exosomes (Exo CTR). (**b**) Cells treated with free AO (200 ng/mL), demonstrating minimal and heterogeneous intracellular signal. (**c**) Cells treated with AO-loaded *Citrus Sinensis*-derived exosomes (Exo-AO) at an equivalent dose (200 ng/mL). The robust and widespread green fluorescence confirms the high delivery efficiency of the exosomal carrier. Images represent a merge of DIC and fluorescence channels. Scale bars: 50 µm.

Confocal laser scanning microscopy (CLSM) distinguishes between non-specific membrane adsorption and real cellular internalization of molecules, including AO. Optical sectioning confirmed the successful translocation of the AO cargo into the cytosolic compartment and its accumulation within perinuclear regions, supporting the hypothesis that PDEVs may represent an efficient delivery system for external molecules. Confocal microscopy analysis demonstrated a marked difference in the intracellular delivery efficiency between free AO and Exo-AO (Figure 4). Cells treated with unloaded *Citrus Sinensis*-derived exosomes (Exo CTR) (Figure 4a) exhibited typical spindle-shaped or dendritic-like melanoma morphology with no detectable basal fluorescence. Treatment with free AO at 200 ng/mL (Figure 4b) resulted in a negligible and stochastic intracellular signal, suggesting that at this sub-micromolar concentration, passive diffusion across the plasma membrane is insufficient for significant drug accumulation. Conversely, Mel501 cells treated with AO-loaded *Citrus Sinensis*-derived exosomes (Exo-AO) (Figure 4c) exhibited a marked and ubiquitous increase in green fluorescence throughout the entire cell population. The fluorescent signal was uniformly distributed within the cytoplasm and perinuclear regions, providing visual evidence of highly efficient, vesicle-mediated internalization. Importantly, the Exo CTR treatment did not induce morphological alterations or signs of cytotoxicity, confirming the biocompatibility of the plant-derived delivery system. An AO concentration of 200 ng/mL was selected to ensure a high signal-to-noise ratio for confocal imaging, thereby providing a clear analytical window to evaluate the higher exosome-mediated carrier effect. It is important to emphasize that the fluorescence analysis is, by definition, a qualitative approach that does not provide a real quantitative evaluation. However, the differences in fluorescence intensity between the cells treated with free-AO (Figure 4b) and those treated with the Exo-AO (Figure 4c) are sufficiently clear.

### 3.5. Induction of AO-Mediated Cell Death

**Figure 5:**
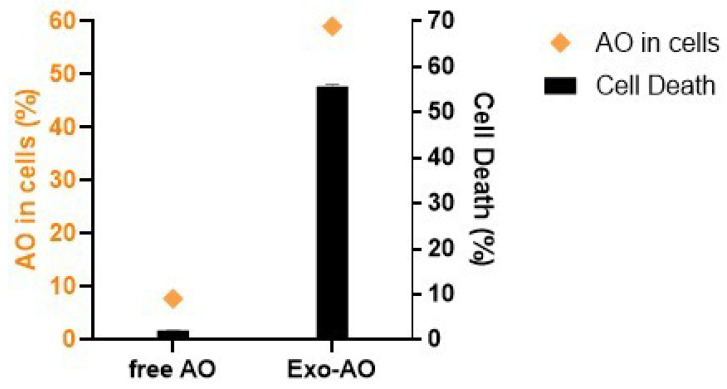
**Comparison between free Acridine Orange and *Citrus Sinensis* exosome-loaded Acridine Orange in Mel501 cells.** Exosome-mediated delivery increased intracellular fluorescence and resulted in higher AO-induced cell death compared to free AO, indicating more efficient cellular uptake of encapsulated AO. In orange are reported the data related to AO signals in cells, in black the percentage of Cell Death.

This set of experiments was aimed at identifying the most efficient loading protocol in the melanoma cellular model and were performed in order to correlate the Acridine Orange fluorescence signal of the resulting cell death. Fluorescence signals were calculated as percentages compared to the AO concentration used for the treatment. As shown in Figure 5, starting from the same amount of AO, the fluorescent signal of the drug detected in cells was much higher after the treatment with Exo-AO (*p* < 0.0001), compared to naked AO, supporting the assumption that the molecule contained within the exosome is able to enter the target cell more easily than the naked molecule. Simultaneously, we evaluated the percentage of cell death with the two different treatments, compared to untreated control cells. Due to the chemical features of AO (i.e., the property of being activated by blue light to obtain the tumoricidal effect), we exposed the human melanoma cell culture to blue light under the microscope and then evaluated cell-death level comparing the cells treated with either Exo-AO and the free AO. The levels of cell death were clearly dependent on the amount of AO detected in the cells, and was extremely more significant in the cells treated with the Exo-AO (*p* < 0.0001).

## 4. Discussion

There is a general and accepted agreement on the need for natural products that should enhance the efficacy of therapeutic compounds with only minimum adverse effects. This urgency in medicine includes delivery systems for therapeutic molecules as well.

The nanotechnological approach has produced a series of nanomaterials that, while promising in delivering therapeutic molecules in the preclinical tests, have limited clinical application, due to their potential toxicity, together with cost-effectiveness.

Previous reports on human extracellular vesicles offered relevant data showing that they are able to deliver a series of therapeutic molecules including chemical drugs [23,24], nanomaterials [25] and therapeutic antibodies [26]. In the last decade, increasing interest has emerged on plant-derived extracellular vesicles (PDEVs). In this sense plant-derived exosomes have convincing advantages as drug deliverers due to their large-scale production, biocompatibility, and ability to efficiently transport therapeutic drugs across cellular barriers. It is important to consider the safety of the source from which the exosomes are derived. Our vesicles are obtained from organic agriculture, thus overcoming the ability of extracellular vesicles to concentrate the pesticides and microbicides used in the intensive farming [19].

In this study we have explored the ability of exosomes from different plants to deliver therapeutic molecules. We have previously shown that human exosomes are able to deliver theranostic molecules such as Acridine Orange (AO) [21]. Unfortunately, human exosomes are not released for their therapeutic use—on the one hand for their potential toxicity, and on the other hand, they are not in fact scalable for industrial application. On the basis of the above reasons and from our previous experience, we decided to explore the possibility of using PDEVs for delivering a traceable molecule such as AO. First of all, we screened PDEVs in three different crops *Citrus Sinensis* (orange), *Citrus Paradisi* (Grapefruit) or *Citrus Limon* (Lemon). These preliminary experiments suggested that we should use exosomes from *Citrus Sinensis* (orange) inasmuch they showed the highest colloidal stability for nano delivery, as compared to extracellular vesicles purified from either *Citrus Paradisi* (Grapefruit) or *Citrus Limon* (Lemon). Our study demonstrated that AO can be efficiently uploaded into exosomes from *Citrus Sinensis*. Preclinical and clinical studies have shown that free AO, while extremely effective against cancer, may have intrinsic toxicity when administered systemically. Thus, we figured out a novel and effective strategy involving the use of exosomes as AO carriers. In our study, we show that plant-derived exosomes could be successfully uploaded with AO through a specific protocol of electroporation. We first performed in situ fluorescence analysis that suggested that (i) plant exosome per se did not induce morphological changes in treated cells and (ii) that Exo-AO led to a clear increase in the uploading of AO within target cells as compared to free-AO. Of course, quantitative evaluation of this phenomenon will be included in future studies. While the data on impedance indirectly support the efficiency of electroporation in loading AO in plant-derived exosomes, The previous literature suggests that the application of an external electric field induces the formation of hydrophilic pores within the lipid bilayer, whose density and stability regulate the mass transfer of the drug across the biological barrier [27,28]. Here we show a drastic reduction in impedance beyond the 1 µg/mL AO threshold, suggesting a saturation of intra-vesicular binding sites or, more likely, the establishment of an electrochemical equilibrium between the intra- and extra-vesicular compartments. Based on previous reports, it appears conceivable that AO may act as an ionic tracer that enhances the global conductivity of the system once the dielectric barrier of the membrane is compromised [29]. The plateau observed at elevated concentrations (> 10 µg/mL) indicates that the system has reached its maximum electroporation-dependent loading capacity, dictated by the finite internal volume and the intrinsic steric constraints of the exosomes [30,31]. However, it appears conceivable that the ionic conductivity of Acridine Orange may contribute to the observed impedance changes.

From a statistical perspective, the pronounced reduction in experimental variance at saturation levels is particularly noteworthy. This phenomenon suggests that while electroporation is a stochastic event at the single-pore level, it yields an extremely uniform collective response when applied to dense vesicular populations. Plant-derived exosomes exhibit superior structural resilience compared to synthetic liposomal systems, likely due to the complexity of their native lipid and protein profiles, which facilitate controlled re-sealing kinetics [32].

In summary, these data confirm that plant-derived exosomes represent a conceivable platform for drug delivery. The strong correlation between cargo concentration and impedance variation supports the use of the latter as a label-free monitoring parameter to validate encapsulation efficiency in real-time [33]. Impedance was used as a label-free physical readout of membrane permeabilization, not as a mechanistic descriptor. Further mechanistic studies will be required to dissect the biophysical basis of this transition. This opens potentially new avenues for the standardization of loading protocols on an industrial scale.

We compared the efficiency of the AO uploading into the target cells between AO loaded into the exosomes and free AO. Importantly, Exo-AO increased the delivery of AO into the target cells. This resulted in a significantly greater cytotoxic effect against tumor cells. The increased intracellular fluorescence observed in the Exo-AO group indicates that the exosomal cargo is not only protected from the extracellular environment but it is also efficiently released into the target cell’s cytoplasm. This higher uptake may be due to the specific lipidomic and proteomic profile of *Citrus*-derived exosomes, which appear to facilitate interaction with the human melanoma cell surface. These findings are consistent with the previous impedance data, confirming that the physical loading efficiency achieved through electroporation may translate into a biological effect. Thus, plant exosomes emerge as a highly effective and biocompatible new approach for oncological drug delivery, potentially reducing the required systemic dosage and minimizing off-target effects.

An important advantage of this approach is that AO, an excitable molecule that may be selectively activated through the exposure to light having the appropriate wavelength (e.g., green fluorescence or x-rays), may increase the selectively of the treatment once uploaded into the exosomes. In fact, we have shown here that excitation with a blue light, following Exo-AO uploading into target cells, has led to a significant increase in the cytotoxic effect against human melanoma cells.

As shown for human EVs, exosomes uploaded with AO are efficiently uploaded into target cells. There are some possible mechanisms underlying this phenomenon, including lipid–lipid interaction, electrostatic interaction or transmembrane active transport. As shown for human vesicles, lipid–lipid interaction leads to membrane–membrane fusion, and this is highly dependent on the electrostatic charges of the interacting membranes. AO represents an ideal model of a theranostic molecule, being at the same time a tracer (naturally fluorescent) and a highly cytotoxic molecule following light stimulation. In fact, the use of AO against malignant tumors was included between the so-called “photodynamic therapies “ [6]. This is clearly an advantage inasmuch as it may allow the induction of tumor cytotoxicity only when the drug (i.e., AO) is delivered to the tumor site, through external photostimulation. The novelty of our study lies in the scientific demonstration that plant-derived extracellular vesicles deriving from organic farming are fully able to be uploaded with AO and to deliver AO into human tumor cells, inducing a marked cell death. Moreover, it deserves to be emphasized that exosomes, which have shown both a high level of biocompatibility and low immunogenicity, may represent a new class of potential therapeutic agents through their natural content of bioactive molecules [18].

It is mandatory that, although here we have shown the ability of exosomes to deliver therapeutic/diagnostic molecules, this approach should be compared to both artificial nanoparticles and human EVs. As far as artificial nanoparticles are concerned, their efficiency in delivering drugs—as compared to the free drugs—has been investigated in some clinical trials [34] that to date did not dispel the existing doubts about their efficacy and toxicity. We have previously investigated the ability of human EVs to deliver both theranostic molecules [23] and nanoparticles [25], showing that in fact they are fully able to be uploaded and transport any type of molecule: However, human EVs are not scalable for an industrial use and have some potential problem in resulting entirely safe [35,36].

Here we provide proof of concept for a new and natural mechanism of drug delivery. It is also potentially safer than the use of both human exosomes and the free molecule; however, it is mandatory that obtaining the scientific evidence for a safer in vivo use requires future in vivo studies. The use of AO also introduces the use of an external control of the therapeutic activation of the drug through photostimulation.

Future studies including vesicle-free controls and frequency-resolved impedance analyses are needed in order to better identify the vesicle-specific action both in the cargo delivery and its downstream biological effects.

## 5. Conclusions

With this study we have shown that plant-derived exosomes from organic crops may be uploaded with a theranostic molecule, such as Acridine Orange, and that within PDEVs, AO exerts its full activity against target cells. The novelty of this report lies in the demonstration that PDEVs from organic crops are fully able to deliver molecules with a double activity as both therapeutic drug and a tracer, also suggesting that they can be used in improving both therapeutic protocols and in promoting new diagnostic strategies. Previous studies on plant-derived exosomes did not use theranostic molecules. We also provide data on the zeta potential of the plant exosomes included in the study that induced us to use the exosomes from orange as the most suitable to be used in delivering molecules. In fact, orange exosomes showed a more stable zeta potential, with values equal to −20.9 ± 1.9 mV compared to both grapefruit (−10.9 ± 1.7 mV), and lemon (−13.58 ± 1.4 mV), that were consistent with their phospholipid content. Thus, we identified orange vesicles as the most suitable for the use in drug delivery, suggesting that the use of zeta potential measurement may help in the choice of the most suitable PDEV source for drug delivery. In fact, for a definitive choice of the most suitable plant-derived extracellular vesicles for drug delivery, additional studies are needed. Another important novelty of our study is the use of a specific protocol of electroporation for the best drug uploading into exosomes.

From a clinical perspective, a successful translation of exosomes to the clinical site requires further standardization and the addressing of some of the significant challenges associated with their reproducible manufacture. This also requires a better understanding of scalable production of the plant-derived exosomes as well.

Of course, this an in vitro study and in vivo studies aimed at defining the body distribution of PDEVs and their potential targeting to different organs and compartments are needed. It is also important to compare PDEVs to mammalian EVs in their ability to drive communication and interaction between cells within a whole body, in order to understand more on the ability of PDEVs to support a proper immune reaction against tumors and infectious agents [37].

This study was intentionally designed as a focused proof-of-concept to evaluate the ability of PDEVs to deliver a molecule that may represent a model of both diagnostic and therapeutic potential. Broader toxicological assessments, including healthy cells and in vivo models, will be addressed in dedicated future studies.

These findings support the potential of plant-derived nanovesicles as a scalable, biocompatible and low-toxicity platform for the delivery of theranostic molecules.

## 6. Patents

The authors declare that this work is related to the European patent application WO2022/201066, entitled “Method of applying electrical impulses for the purpose of loading various molecules into plant-derived nanovesicles.” [38].

## Figures and Tables

**Table 1:** Summary of size (mean and mode) of plant-derived exosomes isolated from the different sources.

Fruit Source	Mean (nm)	Mode (nm)
Grapefruit (*Citrus paradisi*)	164.0 ± 15.4	114.2 ± 8.9
Lemon (*Citrus limon*)	169.9 ± 9.02	126.2 ± 8.4
Orange (*Citrus sinensis*)	158.8 ± 18.7	123.0 ± 8.9

## Data Availability

Data is contained within the article.
